# Supplementary material for: Tailored collagen binding of albumin-fused hyperactive coagulation factor IX dictates in vivo distribution and functional properties
Source: Nat Commun. 2025 Sep 29;16:8433. doi: 10.1038/s41467-025-62955-9 (PMC12480940; doi:10.1038/s41467-025-62955-9)
Supplement: Supplementary file 1 — Supplementary Information [file 41467_2025_62955_MOESM1_ESM.pdf]

SUPPLEMENTARY INFORMATION

Tailored collagen binding of albumin-fused hyperactive coagulation factor IX dictates in vivo distribution and functional properties

Kristin Hovden Aaen, Maria Francesca Testa, Jeannette Nilsen, Rebecca Tarantino, Cesare Canepari, Mascia Benedusi, Sopisa Benjakul, Mari Nyquist-Andersen, Marie Leangen Herigstad, Alessio Cantore, Giuseppe Valacchi, Inger Sandlie, Francesco Bernardi, Mirko Pinotti, Alessio Branchini*, and Jan Terje Andersen*

corresponding authors.

<u>Supplementary Tables:</u>

1. Two-tailed, unpaired Student's t-test with 95% confidence level performed on protein concentrations in samples from the uptake and recycling steps in HERA.

2. Two-tailed, unpaired Student's t-test with 95% confidence level performed on protein concentrations in plasma 24 hours post-administration to Tg32 mice (n = 5).

3. Two-tailed, unpaired Student's t-test with 95% confidence level performed on plasma half-life values from Tg32 mice (n = 5).

4. Two-tailed, unpaired Student's t-test with 95% confidence level performed on protein concentrations in plasma 24 hours post-administration to FIX^{plus} (Balb/c) mice (n = 5).

5. Two-tailed, unpaired Student's t-test with 95% confidence level performed on plasma half-life values from FIXplus (Balb/c) mice (n = 5).

6. Two-tailed, unpaired Student's t-test with 95% confidence level performed on plasma protein concentrations in plasma 1, 24, and 96 h post-administration to HB mice (n = 4 for all groups except n = 5 for $Padua_{KR}$-HSA_{QMP}).

7. Two-tailed, unpaired Student's t-test with 95% confidence level performed on plasma half-life values from HB mice (n = 4 for all groups except n = 5 for $Padua_{KR}$-HSA_{QMP}).

8. Two-tailed, unpaired Student's t-test with 95% confidence level performed on protein concentrations in organ homogenates from HB mice (n = 4 for all groups except n = 5 for $Padua_{KR}$-HSA_{QMP}).

9. Two-tailed, unpaired Student's t-test with 95% confidence level performed on FIX activity levels in plasma 1, 24, and 96 h post-administration to HB mice (n = 4 for all groups except n = 5 for $Padua_{KR}$-HSA_{QMP}).

10. Two-tailed, unpaired Student's t-test with 95% confidence level performed on specific activities of FIX-HSA fusion variants in plasma samples from HB mice, calculated as the mean of four time points (n = 4 for all groups except n = 5 for $Padua_{KR}$-HSA_{QMP}).

11. Two-tailed, unpaired Student's t-test with 95% confidence level performed on functional half-life of FIX-HSA fusion variants in plasma from HB mice (n = 4 for all groups except n = 5 for $Padua_{KR}$-HSA_{QMP}).

12. Contribution of engineering steps performed in FIX-HSA fusion proteins on their PK profile when studied in different mouse models.

13. Prediction of T cell (MHC) epitopes of FIX- and HSA-containing proteins using NetMHC4.1.

14. Prediction of B cell epitopes of FIX- and HSA-containing proteins using the IEDB analysis tool Bepipred Linear Epitope Prediction 2.0.

Supplementary Figures:

1. Representative sensorgrams from SEC performed on affinity purified FIX-HSA fusion proteins and unfused HSA_{WT} and HSA_{QMP}.

2. Non-reducing SDS-PAGE of purified FIX-HSA variants.

3. Functional properties of FIX variants in unfused and HSA-fused formats.

4. Time-course evaluation of albumin detachment from FIX-HSA fusions at the cleavable linker site upon cleavage by FXIa.

5. *In vitro* phospholipid binding of Padua-HSA_{QMP} fusion proteins engineered in position 5 of FIX.

6. Representative sensorgrams from SEC performed on affinity-purified soluble truncated forms of hFcRn and mFcRn.

7. ELISA-based binding assay showing binding between hFcRn or mFcRn and FIX-HSA at pH 7.4.

8. Representative SPR sensorgrams of soluble truncated hFcRn or mFcRn injected over immobilized FIX-HSA at pH 5.5.

9. Representative SPR sensorgrams of soluble truncated hFcRn or mFcRn injected over immobilized FIX-HSA at pH 7.4.

10. Uptake and recycling of FIX-HSA variants studied in HERA.

11. Uptake and recycling of unfused HSA_{WT} and HSA_{QMP} studied in HERA.

12. Plasma concentrations of endogenous mFIX in Tg32, FIX^{plus} (Balb/c), and HB mice.

13. Elimination curves of FIX-HSA fusion proteins in Tg32 mice.

14. Elimination curves of FIX-HSA fusion proteins in FIX^{plus} (Balb/c) mice.

15. IF staining against Col4 on tissues from HB mice.

16. IHC staining against FIX and Col4 on tissues from HB mice.

17. Control IF and IHC staining of tissues from HB mice.

18. Uncropped image of non-reducing SDS-PAGE gel of purified FIX-HSA variants.

19. Uncropped images of Western blots from experiment evaluating albumin detachment from FIX-HSA fusions upon cleavage by FXIa in a time-course.

SUPPLEMENTARY TABLES

Supplementary Table 1. Two-tailed, unpaired Student's t-test with 95% confidence level performed on protein concentrations in samples from the uptake and recycling steps in HERA.

	FIX_{KA}-HSA_{WT}	FIX_{KR}-HSA_{WT}	Padua-HSA_{QMP}	$Padua_{KA}$-HSA_{QMP}	$Padua_{KR}$-HSA_{QMP}
	Uptake<br>(n = 9 for all groups, except n = 8 for FIX_{KA}-HSA_{WT}, FIX_{KR}-HSA_{WT}, n = 6 for $Padua_{KR}$-HSA_{QMP})				
FIX-HSA_{WT}	ns, 0.9515	ns, 0.1016	****, <0.0001	****, <0.0001	****, <0.0001
Padua-HSA_{QMP}	****, <0.0001	****, <0.0001	-	ns, 0.1030	ns, 0.8846
	Recycling<br>(n = 9 for all groups, except n = 7 for FIX_{KA}-HSA_{WT})				
FIX-HSA_{WT}	ns, 0.0960	ns, 0.2664	****, <0.0001	****, <0.0001	****, <0.0001
Padua-HSA_{QMP}	****, <0.0001	****, <0.0001	-	ns, 0.8574	ns, 0.2927

Supplementary Table 2. Two-tailed, unpaired Student's t-test with 95% confidence level performed on protein concentrations in plasma 24 hours post-administration to Tg32 mice (n = 5).

	FIX-HSA_{WT}	FIX_{KA}-HSA_{WT}	FIX_{KR}-HSA_{WT}	$Padua_{KA}$-HSA_{QMP}	$Padua_{KR}$-HSA_{QMP}
FIX-HSA_{WT}		ns, 0.0820	ns, 0.0849	****, <0.0001	***, 0.0009
FIX_{KA}-HSA_{WT}			**, 0.0043	***, 0.0002	**, 0.0015
FIX_{KR}-HSA_{WT}				****, <0.0001	***, 0.0006
$Padua_{KA}$-HSA_{QMP}					ns, 0.1217
$Padua_{KR}$-HSA_{QMP}					

Supplementary Table 3. Two-tailed, unpaired Student's t-test with 95% confidence level performed on plasma half-life values from Tg32 mice (n = 5).

	FIX-HSA_{WT}	FIX_{KA}-HSA_{WT}	FIX_{KR}-HSA_{WT}	$Padua_{KA}$-HSA_{QMP}	$Padua_{KR}$-HSA_{QMP}
FIX-HSA_{WT}		ns, 0.4317	***, 0.0003	***, 0.0009	***, 0.0009
FIX_{KA}-HSA_{WT}			ns, 0.2157	***, 0.0008	***, 0.0008
FIX_{KR}-HSA_{WT}				***, 0.0004	***, 0.0004
$Padua_{KA}$-HSA_{QMP}					ns, 0.9037
$Padua_{KR}$-HSA_{QMP}					

Supplementary Table 4. Two-tailed, unpaired Student's t-test with 95% confidence level performed on protein concentrations in plasma 24 hours post-administration to FIX^{plus} (Balb/c) mice (n = 5).

	FIX-HSA_{WT}	Padua-HSA_{QMP}	$Padua_{KA}$-HSA_{QMP}	$Padua_{KR}$-HSA_{QMP}
FIX-HSA_{WT}		**, 0.0041	***, 0.0006	***, 0.0009
Padua-HSA_{QMP}			ns, 0.6348	ns, 0.2297
$Padua_{KA}$-HSA_{QMP}				ns, 0.1626
$Padua_{KR}$-HSA_{QMP}				

Supplementary Table 5. Two-tailed, unpaired Student's t-test with 95% confidence level performed on plasma half-life values from FIX^{plus} (Balb/c) mice (n = 5).

	FIX-HSA_{WT}	Padua-HSA_{QMP}	$Padua_{KA}$-HSA_{QMP}	$Padua_{KR}$-HSA_{QMP}
FIX-HSA_{WT}		****, <0.0001	****, <0.0001	****, <0.0001
Padua-HSA_{QMP}			ns, 0.0518	ns, 0.3454
$Padua_{KA}$-HSA_{QMP}				ns, 0.1029
$Padua_{KR}$-HSA_{QMP}				

Supplementary Table 6. Two-tailed, unpaired Student's t-test with 95% confidence level performed on plasma protein concentrations in plasma 1, 24, and 96 h post-administration to HB mice (n = 4 for all groups except n = 5 for Padua$_{KR}$-HSA$_{QMP}$).

Protein variant	FIX-HSA$_{WT}$	Padua-HSA$_{QMP}$	Padua$_{KA}$-HSA$_{QMP}$	Padua$_{KR}$-HSA$_{QMP}$
	1 h			
FIX-HSA$_{WT}$		**, 0.0025		
Padua-HSA$_{QMP}$			**, 0.0072	ns, 0.3122
Padua$_{KA}$-HSA$_{QMP}$			-	**, 0.0052
Padua$_{KR}$-HSA$_{QMP}$				
	24 h			
FIX-HSA$_{WT}$		****, <0.0001		
Padua-HSA$_{QMP}$			**, 0.0020	*, 0.0352
Padua$_{KA}$-HSA$_{QMP}$				ns, 0.1052
Padua$_{KR}$-HSA$_{QMP}$				
	96 h			
FIX-HSA$_{WT}$		ns, 0.5126		
Padua-HSA$_{QMP}$		-	ns, 0.7533	ns, 0.1133
Padua$_{KA}$-HSA$_{QMP}$			-	*, 0.0392
Padua$_{KR}$-HSA$_{QMP}$				-

Supplementary Table 7. Two-tailed, unpaired Student's t-test with 95% confidence level performed on plasma half-life values from HB mice (n = 4 for all groups except n = 5 for Padua$_{KR}$-HSA$_{QMP}$).

Protein variant	FIX-HSA$_{WT}$	Padua-HSA$_{QMP}$	Padua$_{KA}$-HSA$_{QMP}$	Padua$_{KR}$-HSA$_{QMP}$
FIX-HSA$_{WT}$		*, 0.0280	***, 0.0008	*, 0.0179
Padua-HSA$_{QMP}$			***, 0.0008	ns, 0.5366
Padua$_{KA}$-HSA$_{QMP}$				**, 0.0052
Padua$_{KR}$-HSA$_{QMP}$				

Supplementary Table 8. Two-tailed, unpaired Student's t-test with 95% confidence level performed on protein concentrations in organ homogenates from HB mice (n = 4 for all groups except n = 5 for Padua$_{KR}$-HSA$_{QMP}$).

Protein variant	FIX-HSA$_{WT}$	Padua-HSA$_{QMP}$	Padua$_{KA}$-HSA$_{QMP}$	Padua$_{KR}$-HSA$_{QMP}$
**Liver**				
FIX-HSA$_{WT}$		ns, 0.5513	***, 0.0002	**, 0.0078
Padua-HSA$_{QMP}$			**, 0.0027	**, 0.0068
Padua$_{KA}$-HSA$_{QMP}$				***, 0.0007
Padua$_{KR}$-HSA$_{QMP}$				
**Kidneys**				
FIX-HSA$_{WT}$		ns, 0.6009	***, 0.0001	***, 0.0002
Padua-HSA$_{QMP}$			**, 0.0018	***, 0.0002
Padua$_{KA}$-HSA$_{QMP}$				****, <0.0001
Padua$_{KR}$-HSA$_{QMP}$				
**Lungs**				
FIX-HSA$_{WT}$		ns, 0.7566	***, 0.0006	**, 0.0017
Padua-HSA$_{QMP}$			****, <0.0001	***, 0.0008
Padua$_{KA}$-HSA$_{QMP}$				****, <0.0001
Padua$_{KR}$-HSA$_{QMP}$				
**Knee joints**				
FIX-HSA$_{WT}$		ns, 0.0520	**, 0.0061	***, 0.0002
Padua-HSA$_{QMP}$			**, 0.0015	**, 0.0013
Padua$_{KA}$-HSA$_{QMP}$				****, <0.0001
Padua$_{KR}$-HSA$_{QMP}$				

Supplementary Table 9. Two-tailed, unpaired Student's t-test with 95% confidence level performed on FIX activity levels in plasma 1, 24, and 96 h post-administration to HB mice (n = 4 for all groups except n = 5 for Padua$_{KR}$-HSA$_{QMP}$).

Protein variant	FIX-HSA$_{WT}$	Padua-HSA$_{QMP}$	Padua$_{KA}$-HSA$_{QMP}$	Padua$_{KR}$-HSA$_{QMP}$
	1 h			
FIX-HSA$_{WT}$		****, <0.0001		
Padua-HSA$_{QMP}$			ns, 0.0630	ns, 0.3483
Padua$_{KA}$-HSA$_{QMP}$				*, 0.0270
Padua$_{KR}$-HSA$_{QMP}$				
	24 h			
FIX-HSA$_{WT}$		****, <0.0001		
Padua-HSA$_{QMP}$			ns, 0.7515	ns, 0.2880
Padua$_{KA}$-HSA$_{QMP}$				ns, 0.4668
Padua$_{KR}$-HSA$_{QMP}$				
	96 h			
FIX-HSA$_{WT}$		**, 0.0094		
Padua-HSA$_{QMP}$			*, 0.124	**, 0.0011
Padua$_{KA}$-HSA$_{QMP}$				****, <0.0001
Padua$_{KR}$-HSA$_{QMP}$				

Supplementary Table 10. Two-tailed, unpaired Student's t-test with 95% confidence level performed on specific activities of FIX-HSA fusion variants in plasma samples from HB mice, calculated as the mean of four time points (n = 4 for all groups except n = 5 for Padua$_{KR}$-HSA$_{QMP}$).

Protein variant	FIX-HSA$_{WT}$	Padua-HSA$_{QMP}$	Padua$_{KA}$-HSA$_{QMP}$	Padua$_{KR}$-HSA$_{QMP}$
FIX-HSA$_{WT}$		****, <0.0001	***, 0.0001	****, <0.0001
Padua-HSA$_{QMP}$			****, <0.0001	ns, 0.5456
Padua$_{KA}$-HSA$_{QMP}$				***, 0.0005
Padua$_{KR}$-HSA$_{QMP}$				

Supplementary Table 11. Two-tailed, unpaired Student's t-test with 95% confidence level performed on functional half-life of FIX-HSA fusion variants in plasma from HB mice (n = 4 for all groups except n = 5 for Padua$_{KR}$-HSA$_{QMP}$).

Protein variant	FIX-HSA$_{WT}$	Padua-HSA$_{QMP}$	Padua$_{KA}$-HSA$_{QMP}$	Padua$_{KR}$-HSA$_{QMP}$
FIX-HSA$_{WT}$		ns, 0.4571	*, 0.0158	***, 0.0008
Padua-HSA$_{QMP}$			ns, 0.1429	***, 0.0009
Padua$_{KA}$-HSA$_{QMP}$				***, 0.0003
Padua$_{KR}$-HSA$_{QMP}$				

Supplementary Table 12. Contribution of engineering steps performed in FIX-HSA fusion proteins on their PK profile when studied in different mouse models.

Mouse model	FcRn expression	FIX expression	Effect on PK	
			HSA$_{QMP}$	FIX$_{K5}$ variants
Tg32	hFcRn	Yes	++++	-
FIX^{plus} (Balb/c)	mFcRn	Yes	++	-/+
HB	mFcRn	No	++	+++

Supplementary Table 13. Prediction of T cell (MHC) epitopes of FIX- and HSA-containing proteins using NetMHC4.1.

Variant	Number of strong binders	Number of weak binders	Number of peptides
WT HSA	5	5	577
QMP HSA	5	4	498
FIX	3	5	407
FIX-WT	8	10	1013
Padua-QMP	9	10	1013
Padua$_{K5A}$-WT	8	11	1013
Padua$_{K5R}$-WT	8	11	1013
Padua$_{K5A}$-QMP	9	10	1013
Padua$_{K5R}$-QMP	9	10	1013
Albutrepenecog alfa (Idelvion, CSL Behring; rFIX-HSA)	8	10	1010
Binding of 9-mer peptides against representative HLA supertypes.<br>Rank threshold for strong binding peptides: 0.5<br>Rank threshold for weak binding peptides: 2.0			

Supplementary Table 14. Prediction of B cell epitopes of FIX- and HSA-containing proteins using the IEDB analysis tool Bepipred Linear Epitope Prediction 2.0.

Variant	# peptides	Peptide sequences
WT HSA	30	KDLGEEN<br>QCPFEDHVKLVNE<br>E<br>AENCDK<br>RETYGE<br>CAKQEPER<br>QHKDDNPNLPRLVRPEVD<br>DNEETF<br>PYF<br>KA<br>RD<br>ASSAK<br>KFGERA<br>KAEFA<br>H<br>LECAD<br>AD<br>EN<br>DSISS<br>EKPLLEKS<br>VENDEMPADLPSLAADFVE<br>YAEAKD<br>RRHPDYSV<br>FDEFKPLVEEPQNLIKQNCELFEQLGEYK<br>KKVPQVSTPTLVE<br>KHPEAKRMPC<br>EKTPVSDR<br>SLVNRRPCFSALEVDETYVPKEFNAETFTFHADICTLSEKERQIKKQT<br>KHKPKATKEQ<br>ETCFAEEGKKLVA
QMP HSA	31	DLGEEN<br>QCPFEDHVKLVNE<br>E<br>AEN<br>K<br>LRETYGE<br>AKQEPER<br>LQHKDDNPNLPRLVRPEV<br>DNEETF<br>HPYF<br>KA<br>ASSAK<br>KFGERA<br>KAEFA<br>H<br>LECAD<br>AD<br>EN<br>DSISS<br>EKPLLEKS<br>VENDEMPADLPSLAADFVE<br>YAEAKD<br>RRHPDYSV<br>FDEFKPLVEEPQNLIKQNCELFEQLGEYK<br>KKVPQVSTPTLVE<br>KHPEAKRMPC<br>EKTPVSDR<br>SLVNRRPCFSALEVDETYVPKEFNAETFTFHAD<br>CTLSEKERQIKK<br>KHKPKATKEQL<br>TCFAEEGKKL

FIX	13	LEEFVQGNLERECMEEKCSFEEAREVFENTERTTEFWKQYVDGDQCESNPCLNGG<br>SCKDDINSYE<br>GFEGKNCELDVTCNIKNGRCEQFCKNSADNK<br>TEGYRLAENQKSCEPAVPFPCGRVSVSQTSKLTRAETVFPDVDYVNSTEAETILDNI<br>TQSTQSFNDFT<br>K<br>EETEHTEQ<br>PHHNYNAAINKYN<br>EYTNIFLKF<br>FHKGRSAL<br>VDRATCLRS<br>KFT<br>GGRDSCQGDS<br>GEECAMKGK<br>VNWIKEK
FIX-WT	39	LEEFVQGNLERECMEEKCSFEEA<br>VFENTERTTEFWKQYVDGDQCESNPCLNGGSCKDDINSYE<br>PFGFEGKNCELDVTCNIKNGRCEQFCKNSADNK<br>TEGYRLAENQKSCEPAVPFPCGRVSVSQTSKLTRAETVFPDVDYVNSTEAETILDNI<br>TQSTQSFNDFT<br>EETEHTEQ<br>PHHNYNAAINKYN<br>EYTNIFLK<br>FHKGRSAL<br>V<br>RATCLRSTKFT<br>GGRDSCQGDS<br>EECAMKG<br>KTKLTSVSQTSKLTRVVGGEDAKPGSDAHKSEVAHRFK<br>L<br>E<br>QCPFEDHVKLVN<br>A<br>ESAEN<br>LRETYGEMADCCAKQEPER<br>LQHKDDNPNLPRLVRPEV<br>PY<br>K<br>A<br>KFGER<br>KAEFA<br>ENQDSISS<br>KPLLEK<br>ENDEMPADLPSLAADFVE<br>RRHPDYSV<br>E<br>KPL<br>E<br>ELFEQLGEYK<br>KKVPQVSTPT<br>HPEAKRMPC<br>EKTPVSDR<br>TESLVNRRPCFSALEVDETYVPKEFNAETFTFHADICTLSEKERQIK<br>PKATKE<br>KK
Padua-QMP	36	LEEFVQGNLERECMEEKCSFEEA<br>VFENTERTTEFWKQYVDGDQCESNPCLNGGSCKDDINSYE<br>PFGFEGKNCELDVTCNIKNGRCEQFCKNSADN<br>TEGYRLAENQKSCEPAVPFPCGRVSVSQTSKLTRAETVFPDVDYVNSTEAETILDNI<br>TQSTQSFNDFT<br>EETEHTEQ<br>PHHNYNAAINKYN<br>YTNIFLK<br>FHKGRSA<br>RATCLL<br>FT<br>GGRDSCQGDSG

		GEECAMKG<br>KTKLTSVSQTSKLTRVVGGEDAKPGSDAHKSEVAH<br>KDLGEE<br>QCPFEDHVKLVN<br>EN<br>LRETYGE<br>EPE<br>QHKDDNPNLPRLVRPEV<br>PY<br>K<br>KFGER<br>KAEFA<br>ENQDSISS<br>EKPLLEK<br>ENDEMPADLPSLAADFVE<br>RRHPDYSV<br>E<br>KPL<br>E<br>ELFEQLGEYK<br>KKVPQVSTPT<br>HPEAKRMP<br>EKTPVSDR<br>TESLVNRRPCFSALEVDETYVPKEFNAQTFTFHADICTLSEKERQIK<br>PKATKE
Padua$_{K5A}$-WT	39	LEEFVQGNLERECMEEKCSFEEA<br>VFENTERTTEFWKQYVDGDQCESNPCLNGGSCKDDINSYE<br>PFGFEGKNCELDVTCNIKNGRCEQFCKNSADNK<br>TEGYRLAENQKSCEPAVPFPCGRVSVSQTSKLTRAETVFPDVDYVNSTEAETILDNI<br>TQSTQSFNDF<br>EETEHTEQ<br>PHHNYNAAINKYN<br>EYTNIFLK<br>FHKGRSA<br>RATCLL<br>T<br>GGRDSCQGDS<br>GEECAMKGK<br>KTKLTSVSQTSKLTRVVGGEDAKPGSDAHKSEVAHRFKDLGEEN<br>QCPFEDHVKLVN<br>E<br>LRETYGE<br>EPE<br>QHKDDNPNLPRLVRPEV<br>D<br>PY<br>K<br>KFGER<br>KAEFA<br>L<br>ENQDSISS<br>KPLLEK<br>ENDEMPADLPSLAADFVE<br>RRHPDYSV<br>E<br>KPL<br>E<br>ELFEQLGEYK<br>KKVPQVSTPT<br>HPEAKRMPC<br>EKTPVSDR<br>T<br>SLVNRRPCFSALEVDETYVPKEFNAETFTFHADICTLSEKERQIK<br>PKATKE<br>KK

Padua$_{K5R}$-WT	38	LEEFVQGNLERECMEEKCSFEE<br>ENTERTTEF<br>KQYVDGDQCESNPCLNGGSCKDDINSYE<br>PFGFEGKNCELDVTCNIKNGRCEQFCKNSADNK<br>TEGYRLAENQKSCEPAVPFPCGRVSVSQTSKLTRAETVFPDVDYVNSTEAETILDNI<br>TQSTQSFNDF<br>EETEHTEQ<br>PHHNYNAAINKYN<br>YTNIFLK<br>FHKGRSA<br>RATCLLS<br>KFT<br>GGRDSCQGDS<br>GEECAMKGK<br>KTKLTSVSQTSKLTRVVGGEDAKPGSDAHKSEVAHRFKDLGEE<br>QCPFEDHVKLVN<br>EN<br>LRETYGE<br>EPE<br>QHKDDNPNLPRLVRPEV<br>PYF<br>K<br>KFGER<br>KAEFA<br>ENQDSISS<br>KPLLEK<br>ENDEMPADLPSLAADFVE<br>RRHPDYSV<br>E<br>KPL<br>E<br>ELFEQLGEYK<br>KKVPQVSTPT<br>HPEAKRMPC<br>EKTPVSDR<br>T<br>SLVNRRPCFSALEVDETYVPKEFNAETFTFHADICTLSEKERQIK<br>PKATKE<br>KK
Padua$_{K5A}$-QMP	37	LEEFVQGNLERECMEEKCSFEE<br>VFENTERTTEFWKQYVDGDQCESNPCLNGGSCKDDINSYE<br>PFGFEGKNCELDVTCNIKNGRCEQFCKNSADNK<br>TEGYRLAENQKSCEPAVPFPCGRVSVSQTSKLTRAETVFPDVDYVNSTEAETILDNI<br>TQSTQSFNDFT<br>EETEHTEQ<br>PHHNYNAAINKYN<br>EYTNIFLK<br>FHKGRSA<br>RATCLL<br>T<br>GGRDSCQGDS<br>GEECAMKGK<br>KTKLTSVSQTSKLTRVVGGEDAKPGSDAHKSEVAH<br>FKDLGEEN<br>QCPFEDHVKLVN<br>E<br>LRETYGE<br>EPE<br>QHKDDNPNLPRLVRPEV<br>PY<br>K<br>KFGER<br>KAEFA<br>L<br>ENQDSISS<br>EKPLLEK<br>ENDEMPADLPSLAADFVE

		RRHPDYSV<br>E<br>KPL<br>E<br>ELFEQLGEYK<br>KKVPQVSTPT<br>HPEAKRMP<br>EKTPVSDR<br>TESLVNRRPCFSALEVDETYVPKEFNAQTFTFHADICTLSEKERQIK<br>PKATKE
Padua$_{K5R}$-QMP	36	LEEFVQGNLERECMEEKCSFEEA<br>VFENTERTTEFWKQYVDGDQCESNPCLNGGSCKDDINSYE<br>PFGFEGKNCELDVTCNIKNGRCEQFCKNSADNK<br>TEGYRLAENQKSCEPAVPFPCGRVSVSQTSKLTRAETVFPDVDYVNSTEAETILDNI<br>TQSTQSFNDFT<br>EETEHTEQ<br>PHHNYNAAINKYN<br>EYTNIFLK<br>FHKGRSA<br>RATCLL<br>T<br>GGRDSCQGDSG<br>GEECAMKGK<br>KTKLTSVSQTSKLTRVVGGEDAKPGSDAHKSEVAH<br>KDLGEE<br>QCPFEDHVKLVN<br>EN<br>LRETYGE<br>EPE<br>QHKDDNPNLPRLVRPEV<br>PY<br>K<br>KFGER<br>KAEFA<br>ENQDSISS<br>EKPLLEK<br>ENDEMPADLPSLAADFVE<br>RRHPDYSV<br>E<br>KPL<br>E<br>ELFEQLGEYK<br>KKVPQVSTPT<br>HPEAKRMP<br>EKTPVSDR<br>TESLVNRRPCFSALEVDETYVPKEFNAQTFTFHADICTLSEKERQIK<br>PKATKE
Albutrepenec og alfa (Idelvion, CSL Behring; rFIX-HSA)	41	LEEFVQGNLERECMEEKCSFE<br>E<br>TERTTEFWKQYVDGDQCESNPCLNGGSCKDDINSYE<br>PFGFEGKNCELDVTCNIKNGRCEQFCKNSADNK<br>TEGYRLAENQKSCEPAVPFPCGRVSVSQTSKLTRAETVFPDVDYVNSTEAETILDNI<br>TQSTQSFNDFT<br>EETEHTEQ<br>PHHNYNAAINKYN<br>EYTNIFLK<br>FHKGRSA<br>VDRATCLRS<br>KFT<br>GGRDSCQGDS<br>GEECAMKGK<br>KTKLTPVSQTSKLTRAETVFPDVDAHKSEVAHR<br>DLGEE<br>QCPFEDHVK<br>AEN<br>LRETYGE<br>EPE

		QHKDDNPNLPRLVRPEV<br>PY<br>K<br>KFGER<br>KAEFA<br>L<br>C<br>ENQDSISS<br>KPLLEK<br>ENDEMPADLPSLAADFVE<br>RRHPDYSV<br>E<br>KPL<br>E<br>ELFEQLGEYK<br>KKVPQVSTPT<br>PEAKRMP<br>EKTPVSDR<br>T<br>SLVNRRPCFSALEVDETYVPKEFNAETFTFHADICTLSEKERQIK<br>PKATKE<br>KK
Threshold: 0.5.		

SUPPLEMENTARY FIGURES

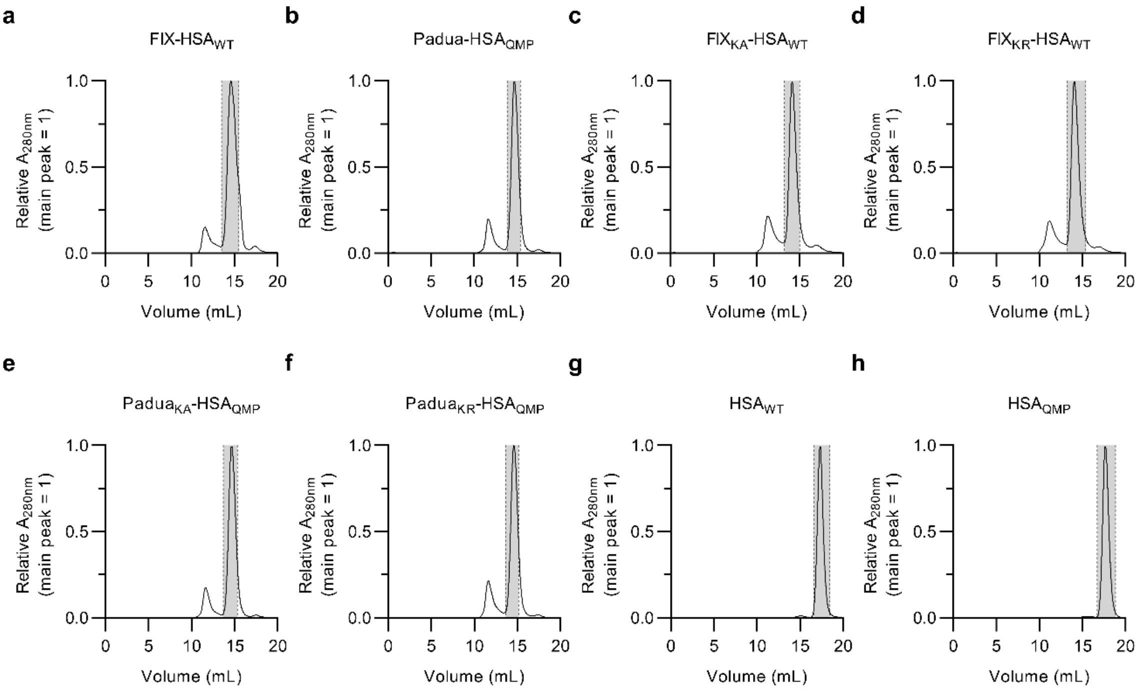

Supplementary Figure 1. Representative sensorgrams from SEC performed on affinity-purified FIX-HSA fusion proteins and unfused HSA$_{WT}$ and HSA$_{QMP}$. Sensorgram from SEC of **a,** FIX-HSA$_{WT}$, **b,** Padua-HSA$_{QMP}$, **c,** FIX$_{KA}$-HSA$_{WT}$, **d,** FIX$_{KR}$-HSA$_{WT}$, **e,** Padua$_{KA}$-HSA$_{QMP}$, **f,** Padua$_{KR}$-HSA$_{QMP}$, **g,** HSA$_{WT}$, and **h,** HSA$_{QMP}$. Data is presented as relative absorbance at 280 nm to that of the main peak (= 1). The shaded areas correspond to the collected fractions.

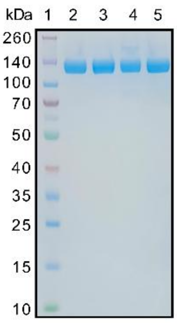

Supplementary Figure 2. Non-reducing SDS-PAGE of purified FIX-HSA variants. The proteins were produced in a serum-free transient Expi293F expression system and purified on an HSA-specific affinity column before SEC to generate monomeric fractions of each protein. A non-reducing SDS-PAGE performed on purified protein fractions confirmed protein integrity and a migration pattern according to their MW (120 kDa). 1: broad range protein ladder (260-10 kDa); 2: FIX$_{KA}$-HSA$_{WT}$; 3: FIX$_{KR}$-HSA$_{WT}$, 4: Padua$_{KA}$-HSA$_{QMP}$; 5: Padua$_{KR}$-HSA$_{QMP}$. An uncropped blot is provided in Supplementary Figure 18.

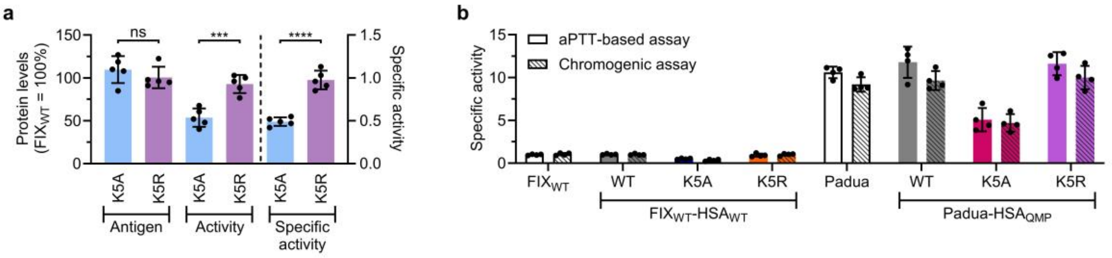

Supplementary Figure 3. Functional properties of FIX variants in unfused and HSA-fused formats. a, Secreted protein and activity levels of unfused FIX (left y-axis), either designed with the K5A (blue) or K5R (light purple) substitution, and the corresponding specific activity (right y-axis). Data represent the group mean ± SD of technical replicates (n =5). Statistical significance was tested by unpaired two-tailed Student's t-tests with 95% confidence level (n = 5; ns = 0.3381, *** $p = 0.0004$, **** $p < 0.0001$). **b,** Specific activity of FIX$_{WT}$ and Padua either unfused or fused to HSA$_{WT}$ or HSA$_{QMP}$, and as WT, K5A, or K5R in position 5 of FIX. Results show the specific activity levels measured by an aPTT-based assay (no pattern) and a chromogenic activity assay (striped). Data represent the group mean ± SD of technical quadruplets.

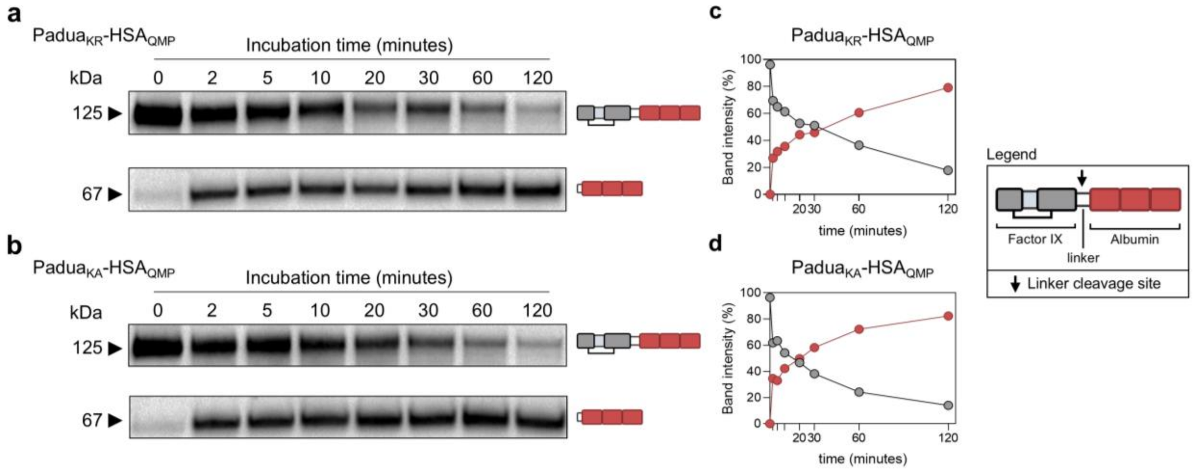

Supplementary Figure 4. Time-course evaluation of albumin detachment from FIX-HSA fusions at the cleavable linker site upon cleavage by FXIa. a-b, Western blotting analysis of **a**, Padua$_{KR}$-HSA$_{QMP}$ or **b**, Padua$_{KA}$-HSA$_{QMP}$ incubated with pdFXIa in a time-course (0-120 minutes). Zymogen fusions (125 kDa) or detached albumin (67 kDa) were detected by polyclonal anti-HSA antibodies. A schematic illustration of the respective protein fragments is shown on the right. **c-d**, Densitometric analysis of band intensity, indicated as % at each time point, of **c**, Padua$_{KR}$-HSA$_{QMP}$ or **d**, Padua$_{KA}$-HSA$_{QMP}$ as zymogen fusion protein (grey circles) or detached HSA (red circles). A schematic illustration of the fusion protein structure with the cleavable site within the linker sequence indicated is shown to the right.

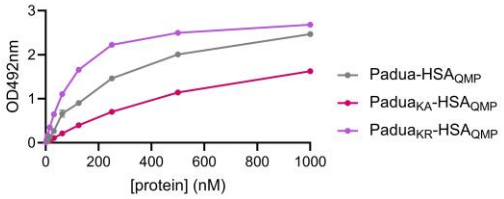

Supplementary Figure 5. In vitro phospholipid binding of Padua-HSA$_{QMP}$ fusion proteins engineered in position 5 of FIX. Results from an *in vitro* ELISA-based phospholipid binding assay where phospholipid vesicles were coated in wells and concentration gradients of Padua-HSA$_{QMP}$ (gray), Padua$_{KA}$-HSA$_{QMP}$ (pink), and Padua$_{KR}$-HSA$_{QMP}$ (purple) were added, before the HSA-region of the fusion proteins was detected by an anti-HSA antibody. The data represent the mean ± SD of technical duplicates.

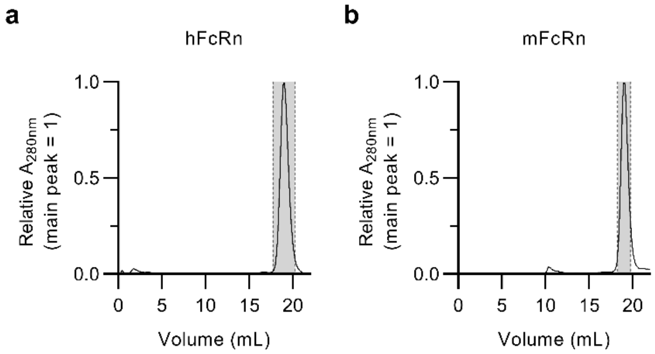

Supplementary Figure 6. Representative sensorgrams from SEC performed on affinity-purified soluble truncated forms of hFcRn and mFcRn. Sensorgram from SEC of **a,** hFcRn and **b,** mFcRn. Data is presented as relative absorbance at 280 nm to that of the main peak (= 1). The shaded areas correspond to the collected fractions.

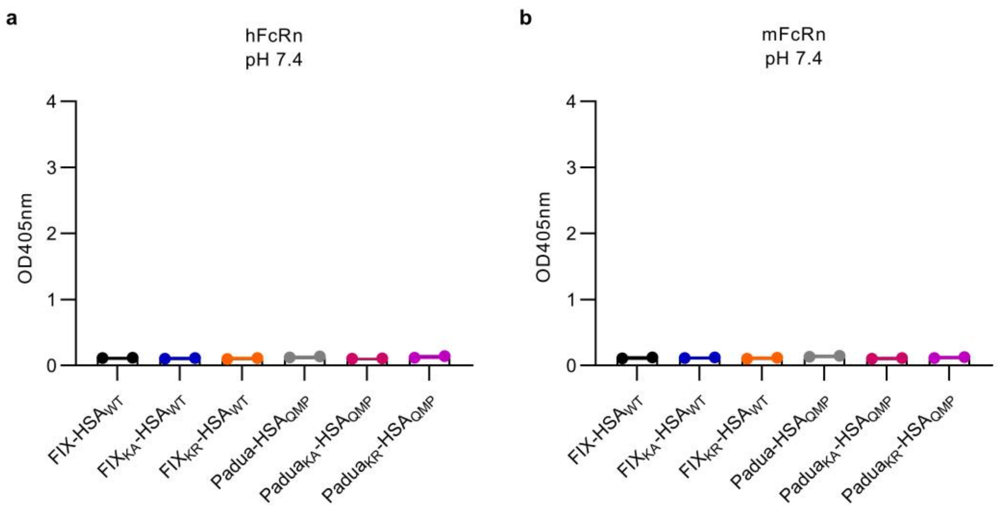

Supplementary Figure 7. ELISA-based binding assay showing binding between hFcRn or mFcRn and FIX-HSA at pH 7.4. His-tagged hFcRn or mFcRn was captured on IgG1 MST/HN coated in the well, before 75 nM FIX-HSA was added to the wells, and the HSA region of the fusion protein was detected by an ALP-conjugated anti-HSA antibody. Binding of FIX-HSA fusion proteins to **a,** hFcRn or **b,** mFcRn at pH 7.4. Data is presented as the mean of technical duplicates from one representative experiment.

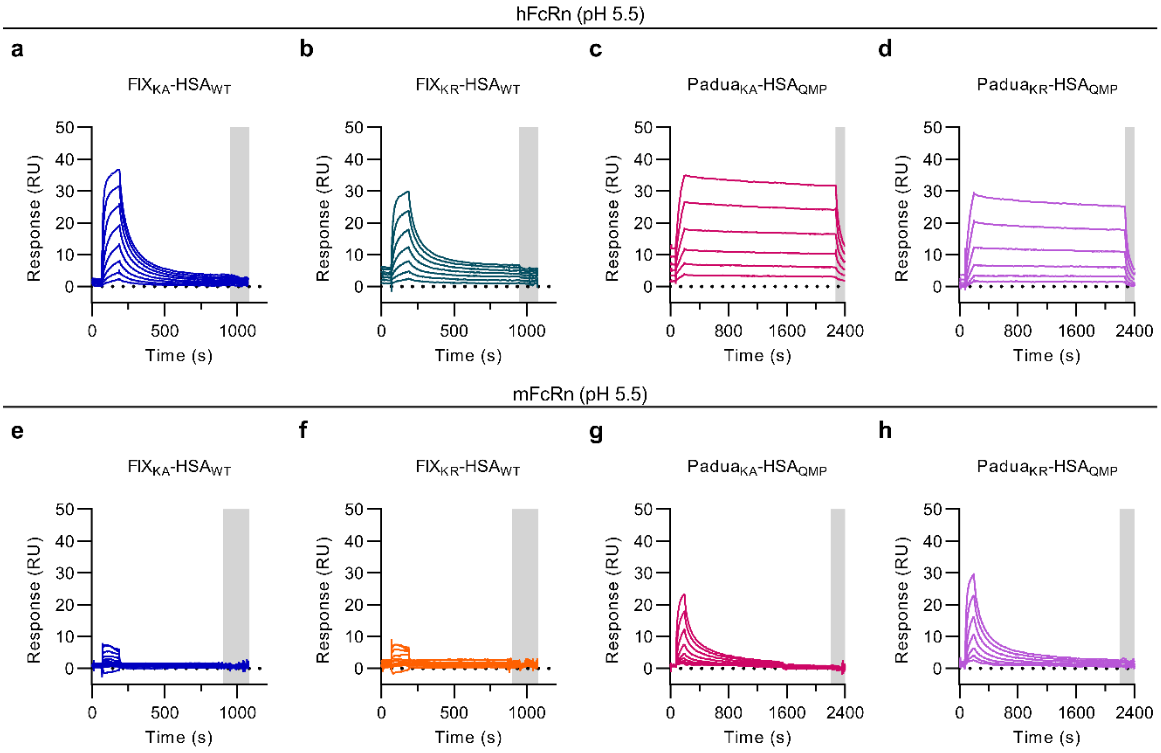

Supplementary Figure 8. Representative SPR sensorgrams of soluble truncated hFcRn or mFcRn injected over immobilized FIX-HSA at pH 5.5. FIX-HSA (200 RU) was immobilized on CM5 sensor chips before hFcRn was injected at **a-b,** 15.6-2000.0 nM over immobilized FIX$_{KA}$-HSA$_{WT}$ and FIX$_{KR}$-HSA$_{WT}$ and **c-d,** 1.95-62.5 nM over immobilized Padua$_{KA}$-HSA$_{QMP}$ and Padua$_{KR}$-HSA$_{QMP}$ at pH 5.5. mFcRn was injected at **e-f,** 7.8-8000 nM over immobilized FIX$_{KA}$-HSA$_{WT}$ and FIX$_{KR}$-HSA$_{WT}$ and **g-h,** 3.9-500 nM over immobilized Padua$_{KA}$-HSA$_{QMP}$ and Padua$_{KR}$-HSA$_{QMP}$ at pH 5.5. Shaded areas represent the regeneration phase (pH 7.4). Data represent one representative run out of three independent runs.

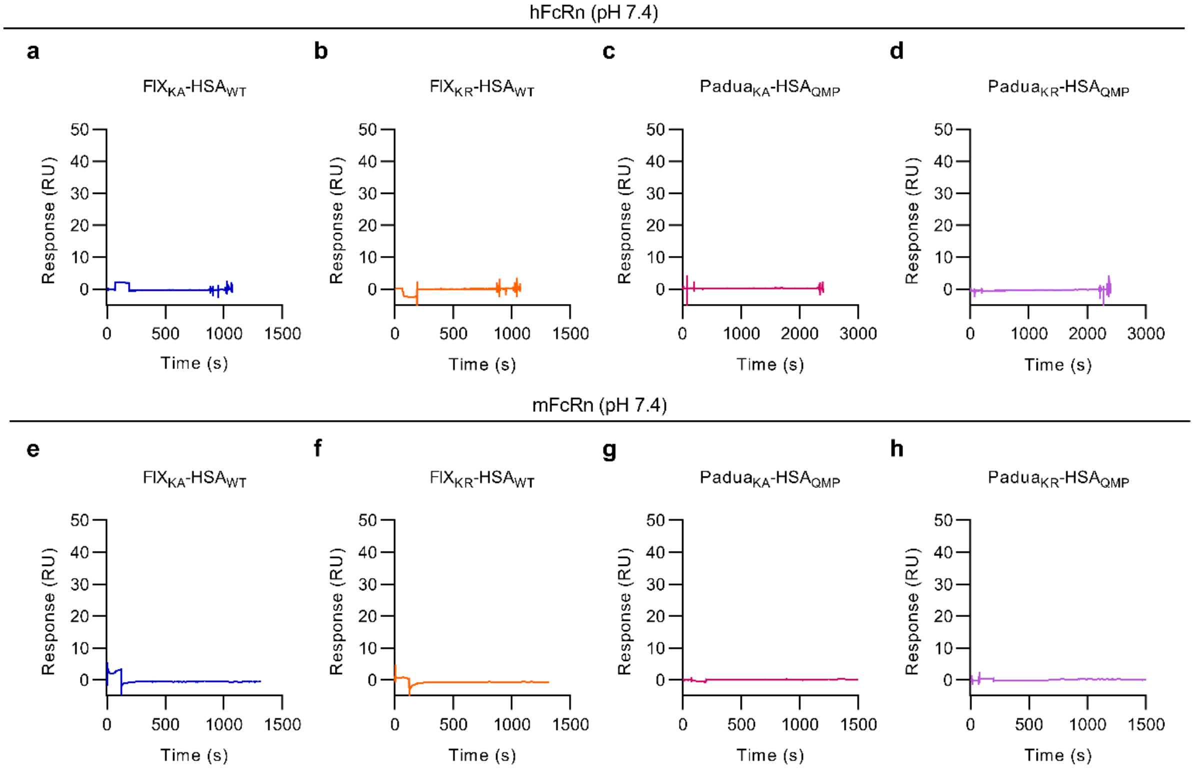

Supplementary Figure 9. Representative SPR sensorgrams of soluble truncated hFcRn or mFcRn injected over immobilized FIX-HSA at pH 7.4. FIX-HSA (200 RU) was immobilized on CM5 sensor chips before hFcRn was injected at **a-b,** 2000 nM over immobilized FIX_{KA}-HSA_{WT} and FIX_{KR}-HSA_{WT} and **c-d,** 62.5 nM of over immobilized $Padua_{KA}$-HSA_{QMP} and $Padua_{KR}$-HSA_{QMP} at pH 7.4. mFcRn was injected at **e-f,** 8000 nM over immobilized FIX_{KA}-HSA_{WT} and FIX_{KR}-HSA_{WT} and **g-h,** 1000 nM over immobilized $Padua_{KA}$-HSA_{QMP} and $Padua_{KR}$-HSA_{QMP} at pH 7.4. Data represent one representative run out of three independent runs.

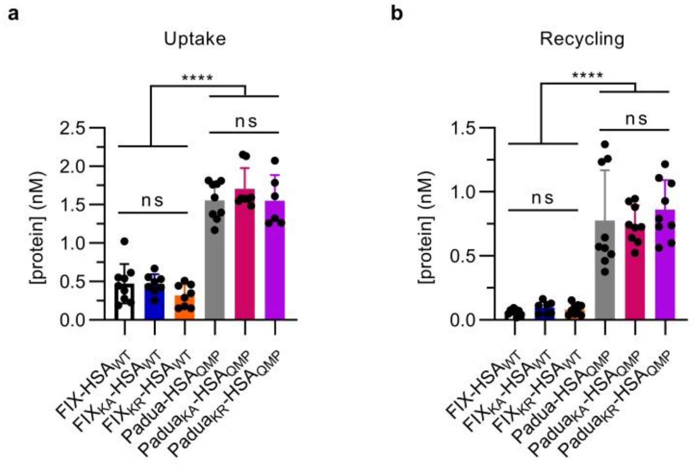

Supplementary Figure 10. Uptake and recycling of FIX-HSA variants studied in HERA. a, Uptake and **b,** recycling of FIX-HSA fusion proteins in HERA presented as protein concentrations. Values represent the group mean ± SD of three independent experiments performed in triplicate. Statistical significance was tested by unpaired two-tailed Student's t-tests with 95% confidence level (uptake: n = 9 except n = n = 8 for FIX_{KA}-HSA_{WT}, FIX_{KR}-$HSA_{WT,}$ and $Padua_{KA}$-HSA_{QMP}, and n = 6 for $Padua_{KR}$-HSA_{QMP}; recycling: n = 9 for all groups; ns, not significant; **** $p < 0.0001$; precise *p*-values are given in Supplementary Table 1). FIX_{KA}-HSA_{WT}, blue; FIX_{KR}-HSA_{WT}, orange; $Padua_{KA}$-HSA_{QMP}, pink; $Padua_{KR}$-HSA_{QMP}, purple; FIX-HSA_{WT}, black; Padua-HSA_{QMP}, gray.

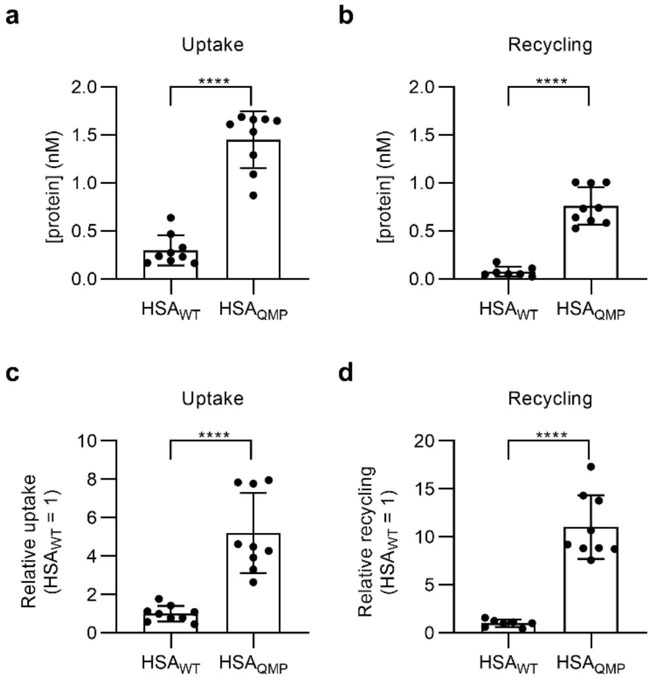

Supplementary Figure 11. Uptake and recycling of unfused HSA_{WT} and HSA_{QMP} studied in HERA. a, uptake and **b,** recycling of unfused HSA_{WT} and HSA_{QMP} in HERA presented as protein concentrations. Values represent the group mean ± SD of three independent experiments performed in triplicate. **c,** Relative uptake compared to HSA_{WT} (= 1). **d,** Relative recycling compared to HSA_{WT} (= 1). Values are presented as the group mean ± SD of three independent experiments performed in technical triplicates (uptake: n = 9; recycling: n = 7 for HSA_{WT}, n = 9 for HSA_{QMP}). Statistical significance was tested by unpaired two-tailed Student's t-tests with 95% confidence level (**** $p < 0.0001$).

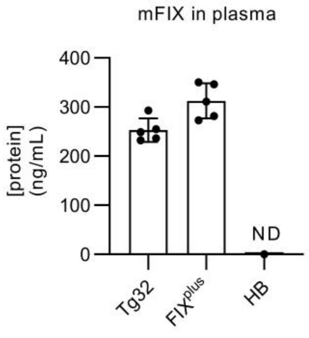

Supplementary Figure 12. Plasma concentrations of endogenous mFIX in Tg32, FIX^{plus} (Balb/c), and HB mice. A mFIX specific ELISA kit was used to quantify endogenous mFIX present in plasma of Tg32, FIX^{plus} (Balb/c), and HB mice. Data are presented as the biological mean ± SD (n = 5, analyzed in technical duplicates). ND, not determined due to signals correlating to background levels in the assay.

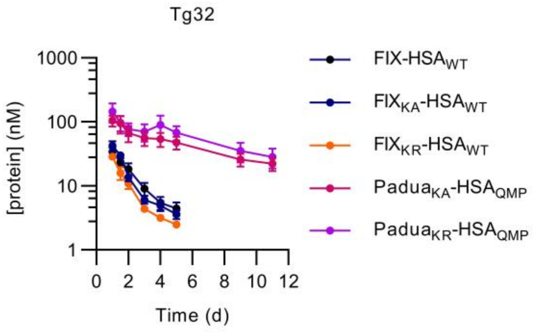

Supplementary Figure 13. Elimination curves of FIX-HSA fusion proteins in Tg32 mice. a) Elimination curves of FIX-HSA fusion proteins composed of FIX either WT in position 5, or containing K5A or K5R, fused to HSA$_{WT}$. **b)** Elimination curves of FIX-HSA fusion proteins composed of FIX Padua either WT in position 5, or containing K5A or K5R, fused to HSA$_{QMP}$. Data is presented as the concentration of protein remaining in plasma over time shown as the biological mean ± SD at each time point (n = 5). FIX-HSA$_{WT}$, black; FIX$_{KA}$-HSA$_{WT}$, blue; FIX$_{KR}$-HSA$_{WT}$, orange*;* Padua$_{KA}$-HSA$_{QMP}$, pink; Padua$_{KR}$-HSA$_{QMP}$, purple.

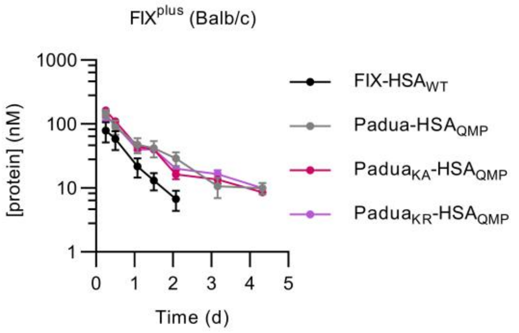

Supplementary Figure 14. Elimination curves of FIX-HSA fusion proteins in FIX^{plus} (Balb/c) mice. Elimination curves of FIX-HSA fusion proteins composed of FIX unmodified in position 5 and fused to HSA$_{WT}$, or containing the Padua substitution and either being unmodified in position 5 or containing K5A or K5R while fused to HSA$_{QMP}$. Data is presented as the concentration of protein remaining in plasma over time shown as the biological mean ± SD at each time point (n = 4-5). FIX-HSA$_{WT}$, black; Padua-HSA$_{QMP}$, gray; Padua$_{KA}$-HSA$_{QMP}$, pink; Padua$_{KR}$-HSA$_{QMP}$, purple.

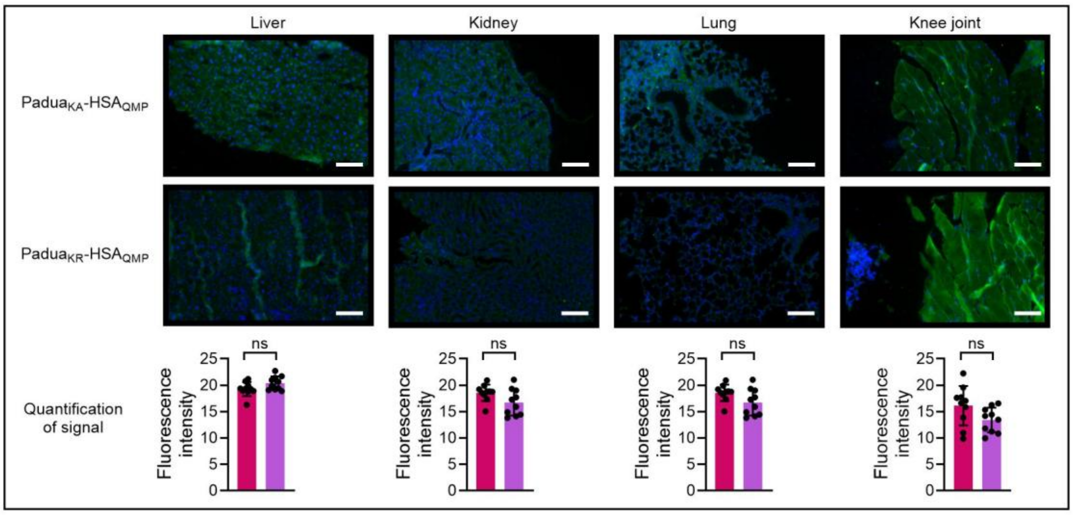

Supplementary Figure 15. IF staining against Col4 on tissues from HB mice. IF staining (green, anti-Col4; blue, DAPI for nuclei) of tissues collected at day 4 from HB mice injected with Padua$_{KR}$-HSA$_{QMP}$ or Padua$_{KR}$-HSA$_{QMP}$ and quantification of the fluorescent signal from ten different fields per image. Statistical significance was tested by unpaired two-tailed Student's t-tests with 95% confidence level (n = 10; ns, not significant; liver, *p* = 0.0771; kidney, *p* = 0.0676; lung, *p* = 0.0676; knee joint, *p* = 0.0668). Padua$_{KR}$-HSA$_{QMP}$, pink; Padua$_{KR}$-HSA$_{QMP}$, purple. Scale bar, 100 µm.

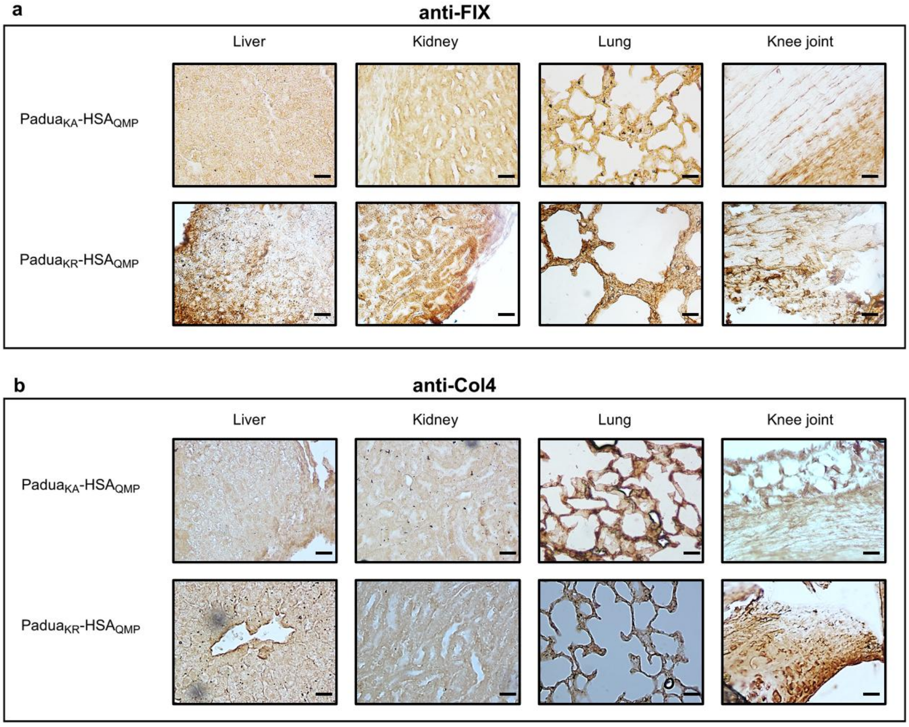

Supplementary Figure 16. IHC staining against FIX and Col4 on tissues from HB mice. Immunohistochemical (3,3′-diaminobenzidine, DAB) staining of tissues from HB mice harvested at day 4 after treatment with Padua$_{KA}$-HSA$_{QMP}$ or Padua$_{KR}$-HSA$_{QMP}$. **a,** Livers, kidney, lungs, and knee joints stained with an anti-human FIX antibody. **b,** Livers, kidney, lungs, and knee joints stained with an anti-mouse Col4 antibody. Scale bar, 50 µm; magnification, 40X.

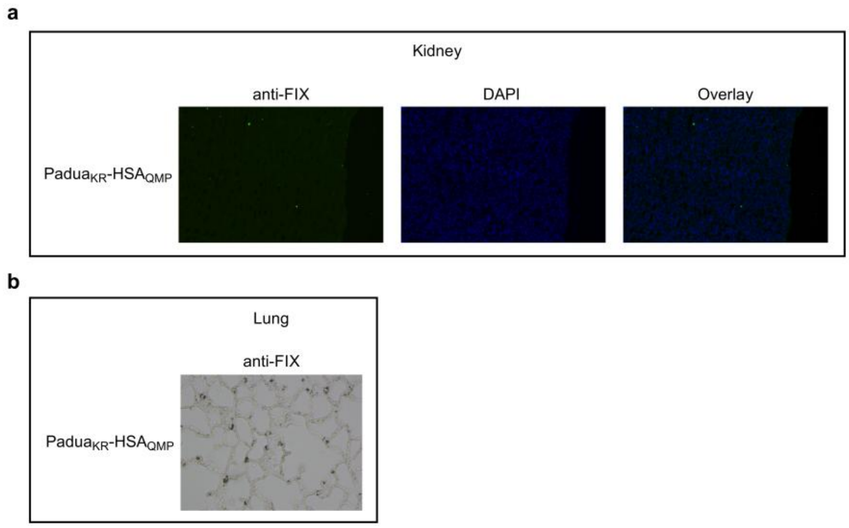

Supplementary Figure 17. Control IF and IHC staining of tissues from HB mice. a, IF staining without primary antibody (goat anti-human FIX) but with secondary antibody (Alexa Fluor 488-conjugated donkey anti-goat antibody; left) or DAPI alone (middle). Images were overlayed (right). **b**, IHC staining without primary antibody (goat anti-human FIX) but with secondary antibody (ImmPRESS (peroxidase) polymer anti-goat IgG reagent). Images show representative tissue from one mouse, administrated with Padua$_{KR}$-HSA$_{QMP}$.

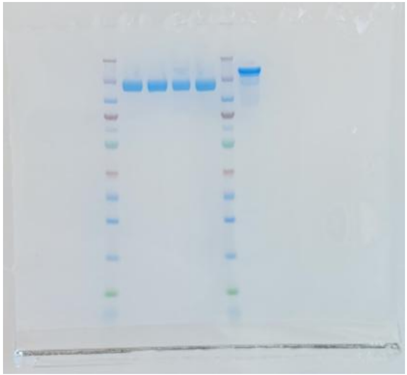

Supplementary Figure 18. Uncropped image of non-reducing SDS-PAGE gel of purified FIX-HSA variants. A non-reducing SDS-PAGE performed on purified protein fractions, representative from >5 independent experiments. Lane 1 (from left): broad range protein ladder (260, 140, 100, 70, 50, 40, 35, 25, 15, and 10 kDa); lane 2: FIX$_{KA}$-HSA$_{WT}$; lane 3: FIX$_{KR}$-HSA$_{WT}$, lane 4: Padua$_{KA}$-HSA$_{QMP}$; lane 5: Padua$_{KR}$-HSA$_{QMP}$, lane 6: broad range protein ladder (same ladder size as in lane 1); lane 7: arbitrary protein.

a

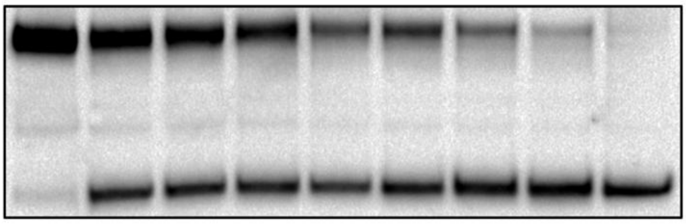

b

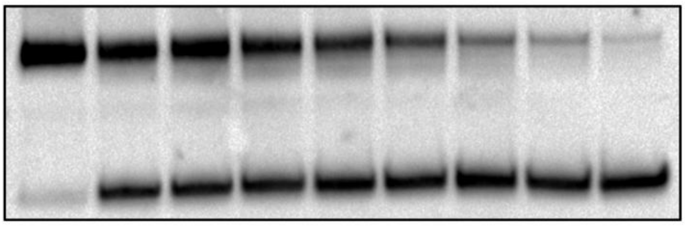

Supplementary Figure 19. Uncropped images of Western blots from experiment evaluating albumin detachment from FIX-HSA fusions upon cleavage by FXIa in a time-course. Western blotting analysis of **a**, $Padua_{KR}$-HSA_{QMP} or **b**, $Padua_{KA}$-HSA_{QMP} incubated with pdFXIa in a time-course, from one experiment. Each lane represent sample at 0, 2, 5, 10, 20, 30, 60, and 120 minutes of incubation. As control for long-time activation, an analysis after 240 minutes was performed (far right), which was not included in Supplementary Figure 4 due to achievement of 80% detachment already at the 120 time point for both fusion proteins. The top row contains bonds corresponding to 125 kDa, the lower row contains bonds corresponding to 67 kDa.
